# Association of high vibration perception threshold with reduced renal function in patients with type 2 diabetes

**DOI:** 10.3389/fendo.2024.1357294

**Published:** 2024-05-30

**Authors:** Yongze Zhang, Biao Zheng, Yimei Li, Ximei Shen, Lingning Huang, Fengying Zhao, Sunjie Yan

**Affiliations:** ^1^ Department of Endocrinology, the First Affiliated Hospital, Fujian Medical University, Fuzhou, China; ^2^ Department of Endocrinology, National Regional Medical Center, the First Affiliated Hospital, Fujian Medical University, Fuzhou, China; ^3^ Clinical Research Center for Metabolic Diseases of Fujian Province, the First Affiliated Hospital, Fujian Medical University, Fuzhou, China; ^4^ Fujian Key Laboratory of Glycolipid and Bone Mineral Metabolism, the First Affiliated Hospital, Fujian Medical University, Fuzhou, China; ^5^ Diabetes Research Institute of Fujian Province, the First Affiliated Hospital, Fujian Medical University, Fuzhou, China; ^6^ Metabolic Diseases Research Institute, the First Affiliated Hospital, Fujian Medical University, Fuzhou, China

**Keywords:** vibration perception threshold, chronic kidney disease, type 2 diabetes, urinary albumin to creatinine ratio, glomerulus, renal tubule

## Abstract

**Objective:**

To investigate the correlation between vibration sensory threshold (VPT) and renal function, including glomerulus and renal tubule, in patients with type 2 diabetes mellitus (T2DM).

**Methods:**

A total of 1274 patients with T2DM who were enrolled in the Department of Endocrinology of the First Affiliated Hospital of Fujian Medical University between January 2017 and June 2020 were included. Patients were grouped according to VPT levels and divided into three groups, including the normal VPT group (VPT<15V), the mild-moderate elevated VPT group (VPT15~25V), and the severely elevated VPT group (VPT≥25 V). Linear correlation analysis was used to analyze the correlation between VPT and renal functions, including glomerulus markers urine microalbumin (MA) and urinary immunoglobulin G (U-IgG), and renal tubule marker α1-microglobulin (α1-MG). Chronic kidney disease (CKD) was defined according to Kidney Disease Improving Global Outcomes (KDIGO) criteria. The binary logistic regression of the relation between VPT and CKD, eGFR<60 ml/min, and UACR >30 mg/g were expressed.

**Results:**

In the mild-moderate and severely elevated VPT group, injury biomarkers of glomerulus (MA and U-IgG), renal tubule (α1-MG), and the incidence of CKD, eGFR<60 ml/min, and UACR > 30 mg/g were gradually increased compared with the normal VPT group. Furthermore, patients with diabetes and severely elevated VPT had significantly higher levels of MA (β=197.54, p=0.042) and α1-MG (β=11.69, p=0.023) compared to those with normal VPT. Also, patients with mild-moderate elevated VPT demonstrate significantly higher levels of MA (β=229.02, p=0.005). Patients in mild-moderate elevated VPT group (OR=1.463, 95% CI 1.005–2.127; OR=1.816, 95% CI 1.212–2.721) and severely elevated VPT group (OR=1.704, 95% CI 1.113–2.611; OR=2.027, 95% CI 1.248–3.294) are at a higher incidence of CKD and elevated levels of UACR>30mg/g compared to those in the VPT normal group. Moreover, the incidence of positive Upro was notably higher in the severely elevated VPT group (OR=1.738, 95% CI 1.182–2.556). However, this phenomenon was not observed in the incidence of eGFR <60 ml/min.

**Conclusion:**

A higher VPT is positively associated with the incidence of CKD in patients with T2DM, particularly with elevated UACR. VPT may serve as a marker for glomerulus and renal tubule injury.

## 1 Introduction

Despite available therapies, chronic kidney disease (CKD) is the leading cause of mortality in diabetic patients, characterized by the progressive decrease in GFR, the incidence of albuminuria, and ultimately irreversible end-stage renal disease (ESRD) (1, 2). However, the optimal timing of treatment may be missed due to the lack of understanding of the potential underlying effect of renal glomerulus and renal tubule on renal function in clinical practice (3). Hence, a systematic assessment of diabetes nephropathy in clinical practice is of utmost importance not only to detect early disease but also to monitor its rate of progression to more advanced disease (4).

Vibratory sensory threshold (VPT) is a vital measure in quantitative sensory examination, primarily indicating lesions in myelinated Aα and Aβ sensory nerve fibers (5). Previous studies have demonstrated that VPT can quickly and accurately identify diabetic peripheral neuropathy (DPN) in clinical settings or primary care, reflecting the impairment of large nerve fibers (6). Due to its non-invasive nature, ease of use, and high patient compliance, VPT examination is increasingly employed in clinical studies of diabetic complications. Recent clinical research has highlighted the clinical significance of VPT in the diagnosis of diabetic peripheral neuropathy (DPN) and diabetic foot ulcers. Additionally, it can serve as an index for assessing cardiac autonomic neuropathy, coronary artery disease, cerebrovascular disease, and hepatic fibrosis in non-alcoholic fatty liver disease among diabetic patients (7, 8). These findings demonstrate the multifaceted clinical applications of VPT. Additionally, the urinary microalbumin (MA) and immunoglobulin G (U-IgG) excretion rates have been widely accepted as markers of glomerular lesions, while α1-microglobulin (α1-MG) possesses the characteristics of a marker for tubular damage (9, 10).

To date, limited research has examined the association between VPT levels and renal dysfunction in patients with T2DM. Additionally, no studies have comprehensively assessed kidney function by evaluating the glomerulus and tubule system (11). The present study investigated the relationship between VPT levels and renal dysfunction, as assessed by glomerulus and tubule in T2DM patients. We anticipate that this research could contribute to identifying novel and promising markers for CKD in individuals with T2DM.

## 2 Materials and methods

### 2.1 Study design

**Figure 1:**
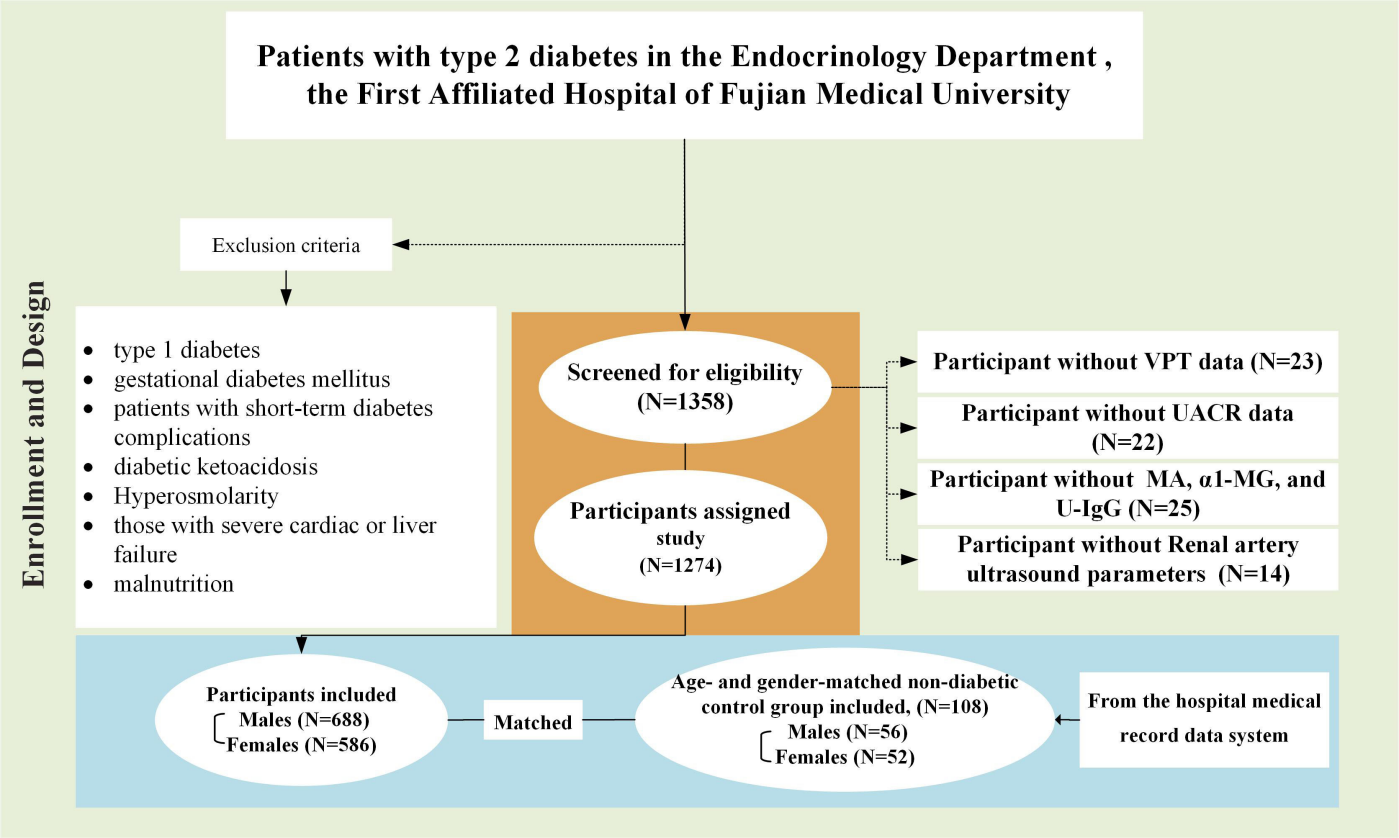
Study design and patient disposition diagram.

This retrospective study enrolled 1274 inpatients with type 2 diabetes (688 male and 586 female subjects) from the First Affiliated Hospital of Fujian Medical University (Fuzhou, China). All participants who met the American Diabetes Association (ADA) diagnostic criteria for type 2 diabetes were included in this study (12, 13). Exclusion criteria encompassed individuals with diabetes other than type 2 diabetes, such as type 1 diabetes or gestational diabetes mellitus. Additionally, patients with short-term diabetes complications, including diabetic ketoacidosis and hyperosmolarity, were excluded. Those with severe cardiac or liver failure, malnutrition, or sarcopenia were also excluded (14). Subsequently, an age- and gender-matched non-diabetic control group was identified in the hospital, comprising 108 subjects. These patients were selected from our medical record data system and were sourced from non-diabetic patients who had previously visited our hospital. The group of ‘non-diabetes comparators (non-DM)’ was used for comparison, as depicted in **Figure 1**.

### 2.2 Clinical and biochemical measurements

Demographic characteristics and medical history were meticulously documented, which included age, sex, duration of diabetes, smoking history, and alcohol intake. Participants’ weight and height were also measured, allowing for the calculation of body mass index (BMI) by dividing weight in kilograms by height in square meters. Systolic blood pressure (SBP) and diastolic blood pressure (DBP) were measured using an automated device after a 15-minute rest period to ensure accuracy.

After a 10-hour fasting period, plasma samples were collected for biochemical indicator analysis. Various parameters were measured, including total cholesterol (TCH), triglycerides (TG), high-density lipoprotein cholesterol (HDL-C), low-density lipoprotein cholesterol (LDL-C), urea nitrogen (BUN), uric acid (UA), and creatinine (Cr). A standard 75-gram oral glucose tolerance test (OGTT) was also performed. The concentration of glycated hemoglobin (HbA1c, measured as a percentage) was determined using high-performance liquid chromatography (VARIANTTM II, BIO-RAD, CA, USA).

Creatinine (Cr) levels were determined using the Benedict-Behre chemical reaction, and the estimated glomerular filtration rate (eGFR) was calculated using the Modification of Diet in Renal Disease (MDRD) formula: eGFR = 186 ×(Cr)^-1.154^ × (age) ^- 0.203^× (0.742 if female). The urinary albumin/creatinine ratio (UACR, MA/Cr) was calculated as the ratio of urinary microalbumin concentration (mg/L) to creatinine concentration (g/L). Early morning urine samples were collected to measure urinary protein (Upro) and specific proteins, including urinary microalbumin (MA), α1-microglobulin (α1-MG), and urinary immunoglobulin G (U-IgG). In this study, the normal ranges for urinary-specific proteins were defined as follows: MA ranged from 0 to 19 mg/L, α1-MG ranged from 0 to 12.5 mg/L, and U-IgG ranged from 0 to 8 mg/L. Calculating the urinary-specific protein to creatinine ratio serves as a modified estimation of the excretion rate and can help mitigate differences between men and women to some extent. Therefore, the UACR, i.e., microalbumin/creatinine ratio (MA/Cr), α1-microglobulin/creatinine ratio (α1-MG/Cr), and immunoglobulin G/creatinine ratio (U-IgG/Cr) were also calculated (15). The VPT examinations were conducted by professional technicians using the American Bio-Thesiometer sensory quantification examiner, following the guidelines of the International Working Group on the Diabetic Foot (16, 17). The patients were examined in a reclined position with their eyes closed in a quiet, relaxed environment. The amplitude of the vibration knob gradually increases until it can be perceived by the person being examined, and the voltage value at this point is read three times continuously, and the average of the three times is taken as the VPT result on that side. According to the level of VPT value, 0~15V indicated normal condition, ≥15~25V mild-moderate lesion, and ≥25V severe lesion; 15V and 25V were used as the dividing point: 0~15V for normal VPT group, ≥15~25V for mild-moderate elevated VPT group, ≥25V for severely elevated VPT group (18). This study used the side with high results for relevant data analysis.

### 2.3 Definitions

#### 2.3.1 T2DM

Criteria for T2DM were defined following the guideline for ADA diagnostic criteria as the presence of symptoms of diabetes (polyphagia, polyphagia, polyuria, weight loss) plus random venous plasma glucose of 11.1 mmol/L, or fasting glucose of 7.0 mmol/L, or 2 h glucose load of 11.1 mmol/L, or glycated hemoglobin of 6.5% or higher (12).

#### 2.3.2 Hypertension

Hypertension was defined as systolic blood pressure (SBP) at rest > 140 mmHg or diastolic blood pressure (DBP) at rest > 90 mmHg or use of antihypertensive drug (19).

#### 2.3.3 CKD

According to Kidney Disease: Improving Global Outcomes (KDIGO) criteria (2012), CKD was defined as structural or functional abnormalities of the kidney for > 3 months, including 1) pathologic abnormalities or markers of damage (albuminuria, abnormal urine sediment, tubular-related lesions, histology and imaging abnormality) or history of renal transplant or dialysis with or without a decrease in GFR; 2) an unexplained decrease in GFR (<60 ml/min) for > 3 months (20).

## 3 Statistical analysis

Statistical analyses were performed using the R software, version 4.3.0. Quantitative data were presented as means (standard deviations) or medians (interquartile ranges), and categorical data as proportions. The chi-square test was used for categorical variables, and the ANOVA and nonparametric tests were used for continuous variables. Correlations between glomerular markers (MA and U-IgG), tubular markers (α1-MG), and VPT were established by linear correlation analysis, and binary logistic regression was used to analyze the correlation between different levels of VPT and CKD, eGFR<60 ml/min, and UACR >30 mg/g. Restricted cubic spline was used to flexibly model and visualize the relationship between VPT and CKD, eGFR<60 ml/min, and UACR >30 mg/g, and an average VPT level of 15V served as a reference without adjustment. *P* value < 0.05 represented statistical significance.

## 4 Results

### 4.1 Study population characteristics

As shown in **Table 1**, the abnormal VPT group exhibited a higher UACR (>30mg/g) or a higher detection rate of CKD, irrespective of the presence of DM. Statistically significant differences were observed among the different VPT groups in terms of glomerular markers (MA and U-IgG), tubular marker (α1-MG), and the incidence of chronic kidney disease (CKD), eGFR <60 ml/min, and UACR >30 mg/g, as shown in **Table 2**. Additionally, there were statistically significant differences among the three groups in terms of age, duration of T2DM, family history of diabetes, alcohol consumption, SBP, DBP, HT, TG, BUN, UA, Upro, and medication history, including α-glycosidase inhibitors, TZDs, insulin therapy, β-blockers, calcium channel blockers, ACEI, ARB. However, no significant differences were observed among the three groups with respect to gender, BMI, LDL-C, HDL-C, TCH, FPG, 2hPG, HbA1c, PSV, and medication history, including metformin, sulfonylurea, and glinides.

**Table 1:** Baseline characteristics of patients with and without type 2 diabetes mellitus.

	DM	non-DM	P-value	DM with normal VPT	DM with abnormal VPT	non-DM with normal VPT	non-DM with abnormal VPT	P-value
	(N=1274)	(N=108)		(N=614)	(N=660)	(N=82)	(N=26)	
Age (years)	61.2 (12.4)	62.6 (9.80)	0.493	56.3 (12.8)	65.7 (10.0)	60.3 (9.07)	69.9 (8.43)	<0.001
Male, n (%)	688 (54.0%)	56 (51.9%)	0.667	332 (54.1%)	356 (53.9%)	42 (51.2%)	14 (53.8%)	0.970
VPT, left (V)	16.8 (10.9)	12.0 (7.28)	<0.001	9.28 (3.13)	23.8 (10.9)	9.19 (2.54)	20.7 (10.1)	<0.001
VPT, right (V)	15.9 (11.6)	12.1 (8.08)	<0.001	8.67 (3.00)	22.6 (12.6)	8.73 (3.07)	22.9 (9.52)	<0.001
Diabetes duration (years)	9.68 (7.33)	/	/	8.47 (6.98)	10.8 (7.48)	/	/	/
Family history of diabetes, n (%)	471 (37.0%)	16 (14.8%)	<0.001	266 (43.3%)	205 (31.1%)	13 (15.9%)	3 (11.5%)	<0.001
Smoking, n (%)	338 (26.5%)	11 (10.2%)	<0.001	150 (24.4%)	188 (28.5%)	8 (9.8%)	3 (11.5%)	<0.001
Drinking, n (%)	118 (9.3%)	7 (6.5%)	0.333	48 (7.8%)	70 (10.6%)	6 (7.3%)	1 (3.8%)	0.238
BMI (Kg/m2)	24.6 (3.82)	22.0 (4.43)	<0.001	24.4 (3.72)	24.8 (3.92)	21.8 (4.68)	22.8 (3.48)	<0.001
Hypertension, n (%)	690 (54.2%)	0 (0%)	<0.001	282 (45.9%)	408 (61.8%)	0 (0%)	0 (0%)	<0.001
SBP (mmHg)	136 (19.5)	125 (17.8)	<0.001	133 (18.0)	138 (20.6)	122 (16.1)	133 (21.0)	<0.001
DBP (mmHg)	79.3 (10.8)	74.0 (11.0)	<0.001	79.9 (10.9)	78.8 (10.7)	74.0 (10.3)	74.1 (13.1)	<0.001
FPG (mmol/L)	8.13 (3.66)	4.69 (0.629)	<0.001	8.35 (3.63)	7.92 (3.68)	4.72 (0.63)	4.58 (0.62)	<0.001
2hPG (mmol/L)	12.8 (4.33)	6.60 (2.02)	<0.001	12.8 (4.27)	12.7 (4.40)	6.46 (1.90)	7.04 (2.41)	<0.001
HbA1c (%)	9.15 (2.46)	5.68 (0.400)	<0.001	9.13 (2.51)	9.16 (2.42)	5.69 (0.40)	5.66 (0.41)	<0.001
TG (mmol/l)	1.80 (1.63)	1.23 (0.64)	<0.001	1.90 (1.81)	1.71 (1.43)	1.20 (0.64)	1.31 (0.66)	<0.001
TCH (mmol/l)	4.47 (1.25)	5.20 (1.32)	<0.001	4.54 (1.27)	4.41 (1.22)	5.28 (1.33)	4.95 (1.29)	<0.001
LDL-C (mmol/l)	2.82 (1.03)	3.48 (1.21)	<0.001	2.86 (1.09)	2.77 (0.979)	3.55 (1.22)	3.26 (1.19)	<0.001
HDL-C (mmol/l)	1.09 (0.337)	1.48 (0.351)	<0.001	1.11 (0.330)	1.07 (0.343)	1.51 (0.338)	1.39 (0.383)	<0.001
BUN (mmol/L)	6.06 (2.88)	4.96 (1.13)	<0.001	5.72 (2.38)	6.37 (3.25)	4.93 (0.935)	5.04 (1.61)	<0.001
UA (mmol/L)	327 (97.6)	279 (78.5)	<0.001	320 (91.8)	334 (102)	278 (81.3)	283 (70.6)	<0.001
Cr (umol/L)	70.5 (58.4)	58.7 (10.7)	0.236	65.0 (49.5)	75.5 (65.2)	59.2 (10.8)	57.0 (10.3)	<0.001
MA/Cr (mg/g)	14.82 (6.58-75.19)	5.81 (4.09-12.91)	<0.001	9.73 (5.44-38.74)	26.31 (8.35-125.47)	4.7 (3.99-10.25)	22.64 (9.98-59.52)	<0.001
U-IgG/Cr (mg/g)	8.48 (4.78-28.36)	NA	/	6.45 (4.11-14.39)	11.19 (5.5-39.73)	NA	NA	/
α1-MG/Cr (mg/g)	16.07 (8.69-35.78)	NA	/	12.11 (7.24-22.39)	19.59 (10.42-49.78)	NA	NA	/
UACR>30mg/g	318 (25.0%)	2 (1.9%)	<0.001	108 (17.6%)	210 (31.8%)	0 (0%)	2 (7.7%)	<0.001
eGFR<60 ml/min	93 (7.3%)	1 (0.9%)	0.020	26 (4.2%)	67 (10.2%)	1 (1.2%)	0 (0%)	<0.001
CKD	317 (24.9%)	3 (2.8%)	<0.001	108 (17.6%)	209 (31.7%)	1 (1.2%)	2 (7.7%)	<0.001
Upro	377 (29.6%)	8 (7.4%)	<0.001	156 (25.4%)	221 (33.5%)	6 (7.3%)	2 (7.7%)	<0.001
Metformin, n (%)	764 (60.0%)	/	/	361 (58.8%)	403 (61.1%)	/	/	/
Sulfonylureas, n (%)	445 (34.9%)	/	/	203 (33.1%)	242 (36.7%)	/	/	/
Glinides, n (%)	208 (16.3%)	/	/	85 (13.8%)	123 (18.6%)	/	/	/
α-glucosidase inhibitors, n (%)	551 (43.2%)	/	/	242 (39.4%)	309 (46.8%)	/	/	/
TZDs, n (%)	82 (6.4%)	/	/	52 (8.5%)	30 (4.5%)	/	/	/
Insulin therapy, n (%)	474 (37.2%)	/	/	192 (31.3%)	282 (42.7%)	/	/	/
β-blockers, n (%)	99 (7.8%)	/	/	34 (5.5%)	65 (9.8%)	/	/	/
CCB, n (%)	375 (29.4%)	/	/	150 (24.4%)	225 (34.1%)	/	/	/
ACEI, n (%)	90 (7.1%)	/	/	33 (5.4%)	57 (8.6%)	/	/	/
ARB, n (%)	261 (20.5%)	/	/	106 (17.3%)	155 (23.5%)	/	/	/
Diuretics, n (%)	50 (3.9%)	/	/	21 (3.4%)	29 (4.4%)	/	/	/

NA, not available; Normal VPT, VPT <15V; Abnormal VPT, VPT ≥15V; BMI, body mass index; FPG, fasting plasma glucose; 2hPG, 2-hour postprandial plasma glucose; HbAlc, glycosylated hemoglobin; TG, triglyceride; TCH, cholesterol; LDL-C, low-density lipoprotein cholesterol; HDL-C, high-density lipoprotein cholesterol; BUN, blood urea nitrogen; UA, uric acid; Cr, serum creatinine; MA/Cr, microalbumin/creatinine ratio;U-IgG/Cr, immunoglobulin G/creatinine ratio;α1-MG/Cr, α1-microglobulin/creatinine ratio; UACR, urinary albumin-to-creatinine ratio; eGFR, estimated glomerular filtration rate; CKD, Chronic kidney disease; RI, renal artery resistance index; PSV, peak systolic flow velocity; TZDs, Thiazolidinediones; CCB, calcium channel blockers; ACEI, angiotensin-converting enzyme inhibitors; ARB, angiotensin receptor blockers; /, the absence of corresponding data.

**Table 2:** Baseline characteristics of 1274 patients with type 2 diabetes mellitus stratified by VPT.

	Normal VPT group (n=614)	Mild to moderate elevated VPT group (n=385)	Severely elevated VPT group (n=275)	P-value
Age (years)	56.3 (12.8)	63.9 (10.1)	68.2 (9.38)	<0.001
Male, n (%)	332 (54.1%)	200 (51.9%)	156 (56.7%)	0.478
Diabetes duration (years)	8.47 (6.98)	10.2 (7.37)	11.6 (7.56)	<0.001
Family history of diabetes, n (%)	266 (43.3%)	124 (32.2%)	81 (29.5%)	<0.001
Smoking, n (%)	150 (24.4%)	108 (28.1%)	80 (29.1%)	0.250
Drinking, n (%)	48 (7.8%)	52 (13.5%)	18 (6.5%)	0.002
BMI (Kg/m2)	24.4 (3.72)	24.7 (3.97)	24.8 (3.84)	0.444
Hypertension, n (%)	282 (45.9%)	231 (60.0%)	177 (64.4%)	<0.001
SBP (mmHg)	133 (18.0)	137 (20.9)	139 (20.1)	<0.001
DBP (mmHg)	79.9 (10.9)	79.5 (10.8)	77.7 (10.5)	0.015
FPG (mmol/L)	8.35 (3.63)	8.00 (3.48)	7.82 (3.96)	0.022
2hPG (mmol/L)	12.8 (4.27)	12.6 (4.25)	12.9 (4.60)	0.762
HbA1c (%)	9.13 (2.51)	9.10 (2.31)	9.25 (2.55)	0.752
TG (mmol/l)	1.90 (1.81)	1.78 (1.49)	1.60 (1.34)	0.023
TCH (mmol/l)	4.54 (1.27)	4.47 (1.24)	4.31 (1.18)	0.028
LDL-C (mmol/l)	2.86 (1.09)	2.83 (0.99)	2.68 (0.95)	0.071
HDL-C (mmol/l)	1.11 (0.33)	1.07 (0.28)	1.07 (0.41)	0.052
BUN (mmol/L)	5.72 (2.38)	5.90 (2.47)	7.05 (4.04)	<0.001
UA (mmol/L)	320 (91.8)	333 (99.9)	337 (106)	0.097
Cr (umol/L)	57.00 (46.45-71.00)	60.00 (47.40-75.40)	63.10 (51.60-82.00)	<0.001
MA/Cr (mg/g)	9.73 (5.44-38.74)	21.62 (7.28-103.35)	28.74 (10.16-175.87)	<0.001
U-IgG/Cr (mg/g)	6.07 (3.99-11.68)	7.43 (4.64-21.1)	11.2 (4.7-46.3)	<0.001
α1-MG/Cr (mg/g)	11.8 (6.6-20.18)	14.25 (7.91-25.15)	17.4 (8.32-38.23)	<0.001
UACR>30mg/g	108 (17.6%)	116 (30.1%)	94 (34.2%)	<0.001
eGFR<60 ml/min	26 (4.2%)	33 (8.6%)	34 (12.4%)	<0.001
CKD	108 (17.6%)	107 (27.8%)	102 (37.1%)	<0.001
Upro	156 (25.4%)	111 (28.8%)	110 (40.0%)	<0.001
Metformin, n (%)	361 (58.8%)	249 (64.7%)	154 (56.0%)	0.058
Sulfonylureas, n (%)	203 (33.1%)	139 (36.1%)	103 (37.5%)	0.378
Glinides, n (%)	85 (13.8%)	74 (19.2%)	49 (17.8%)	0.061
α-glucosidase inhibitors, n (%)	242 (39.4%)	183 (47.5%)	126 (45.8%)	0.026
TZDs, n (%)	52 (8.5%)	18 (4.7%)	12 (4.4%)	0.017
Insulin therapy, n (%)	192 (31.3%)	159 (41.3%)	123 (44.7%)	<0.001
β-blockers, n (%)	34 (5.5%)	36 (9.4%)	29 (10.5%)	0.014
CCB, n (%)	150 (24.4%)	132 (34.3%)	93 (33.8%)	<0.001
ACEI, n (%)	33 (5.4%)	25 (6.5%)	32 (11.6%)	0.003
ARB, n (%)	106 (17.3%)	87 (22.6%)	68 (24.7%)	0.018
Diuretics, n (%)	21 (3.4%)	11 (2.9%)	18 (6.5%)	0.037

BMI, body mass index; FPG, fasting plasma glucose; 2hPG, 2-hour postprandial plasma glucose; HbAlc, glycosylated hemoglobin; TG, triglyceride; TCH, cholesterol; LDL-C, low-density lipoprotein cholesterol; HDL-C, high-density lipoprotein cholesterol; BUN, blood urea nitrogen; UA, uric acid; Cr, serum creatinine; MA/Cr, microalbumin/creatinine ratio;U-IgG/Cr, immunoglobulin G/creatinine ratio;α1-MG/Cr, α1-microglobulin/creatinine ratio; UACR, urinary albumin-to-creatinine ratio; eGFR, estimated glomerular filtration rate; CKD, Chronic kidney disease; RI, renal artery resistance index; PSV, peak systolic flow velocity; TZDs, Thiazolidinediones; CCB, calcium channel blockers; ACEI, angiotensin-converting enzyme inhibitors; ARB, angiotensin receptor blockers.

### 4.2 Relationship between VPT and CKD, eGFR <60 ml/min, and UACR >30 mg/g

**Figure 2:**
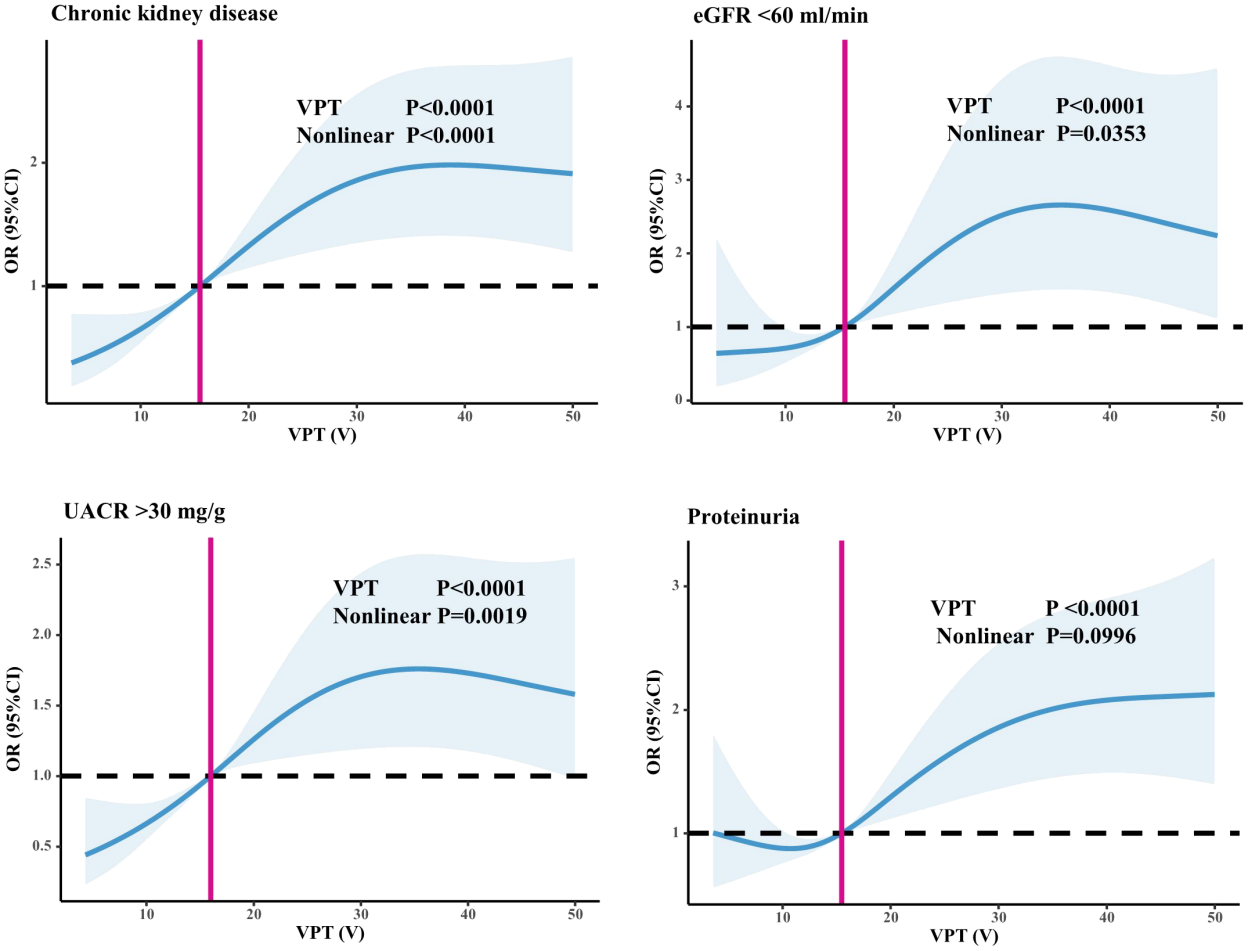
Relationship between VPT and CKD, eGFR <60 ml/min, and UACR >30 mg/g. Restricted cubic splines to flexibly model and visualize the relationship between VPT and CKD, eGFR <60 ml/min, UACR >30 mg/g, and positive Upro. An average VPT level of 15V served as a reference.

As shown in **Table 2**, the incidence of CKD (17.6% vs. 27.8% vs. 37.1%, P=0.000), the proportion of UACR > 30 mg/g (27.7% vs. 43.0% vs. 49.0%, P<0.001), the proportion of eGFR < 60 ml/min (4.6% vs. 9.0% vs. 13.7%, P<0.001), and the proportion of positive Upro (27.3% vs 30.4% vs 45.1%, P<0.001) among the three groups were significantly different. As shown in **Figure 2**, we used restricted cubic splines to flexibly model and visualize the relationship between VPT and CKD, eGFR <60 ml/min, UACR >30 mg/g, and positive Upro. The detection rate of CKD, eGFR <60 ml/min, UACR >30 mg/g was relatively flat until around 15V of VPT, after which it started to rapidly decrease forward and increase afterward (P for nonlinearity <0.05). However, a nonlinear trend was not observed in positive Upro outcomes, suggesting that even when the VPT was < 15V, there could still be differences in the risk of developing CKD, eGFR <60 ml/min, and UACR >30 mg/g.

To investigate further whether VPT is an independent factor associated with CKD in patients with T2DM, we conducted a multifactorial logistic regression analysis, as detailed in **Table 3**. This analysis was performed after adjusting for potential confounding factors, including age, duration of diabetes, family history of diabetes, SBP, DBP, HT, TG, FPG, BUN, UA, Cr, smoking history of alcohol consumption, and medication use history. As expected, the results showed that patients in the VPT mild-moderate elevated group (OR=1.463, 95% CI=1.005–2.127, P=0.047) and VPT severely elevated group (OR=1.704, 95% CI=1.113–2.611, P=0.014) were at a higher risk of CKD compared to those in the VPT normal group. Furthermore, after adjusting for the confounding factors, the risk of UACR >30 mg/g was elevated in both the VPT mild-moderate elevated group (OR=1.816, 95% CI=1.212–2.721, P=0.004) and the VPT severely elevated group (OR=2.027, 95% CI=1.248–3.294, P=0.004), when compared to the VPT normal group. However, no statistically significant differences were found in the incidence of eGFR <60 ml/min among the three VPT groups. Additionally, compared with the VPT normal group, the incidence of positive Upro was notably higher in the VPT severely elevated group (OR=1.738, 95% CI=1.182–2.556, P=0.005).

**Table 3:** Relationship between VPT and CKD, eGFR <60 ml/min, and UACR >30 mg/g.

		Unadjusted		Adjusted	
		OR (95% Cl)	P-value	OR (95% Cl)	P-value
CKD	Normal	Reference	–	Reference	–
	Mild to moderate elevated	1.803 (1.330-2.446)	<0.001	1.463 (1.005-2.127)	0.047*
	Severely elevated	2.762 (2.004-3.808)	<0.001	1.704 (1.113-2.611)	0.014*
eGFR<60ml/min	Normal	Reference	–	Reference	–
	Mild to moderate elevated	2.076 (1.220-3.533)	0.007	13.212 (0.675-258.51)	0.089
	Severely elevated	3.303 (1.935-5.636)	<0.001	1.838 (0.153-22.057)	0.631
UACR>30mg/g	Normal	Reference	–	Reference	–
	Mild to moderate elevated	1.967 (1.418-2.729)	<0.001	1.816 (1.212-2.721)	0.004*
	Severely elevated	2.505 (1.748-3.588)	<0.001	2.027 (1.248-3.294)	0.004*
Upro (+)	Normal	Reference	–	Reference	–
	Mild to moderate elevated	1.163 (0.871-1.552)	0.307	0.994 (0.713-1.387)	0.973
	Severely elevated	2.184 (1.598-2.984)	<0.001	1.738 (1.182-2.556)	0.005*

Adjusted for confounding factors such as age, duration of diabetes, family history of diabetes, SBP, DBP, HT, TG, FPG, BUN, UA, Cr, history of smoking, history of alcohol consumption, and history of medication use; –, the absence of corresponding data.

### 4.3 Linear correlation analysis of VPT with glomerular, tubular, and renal artery damage indicators

**Figure 3:**
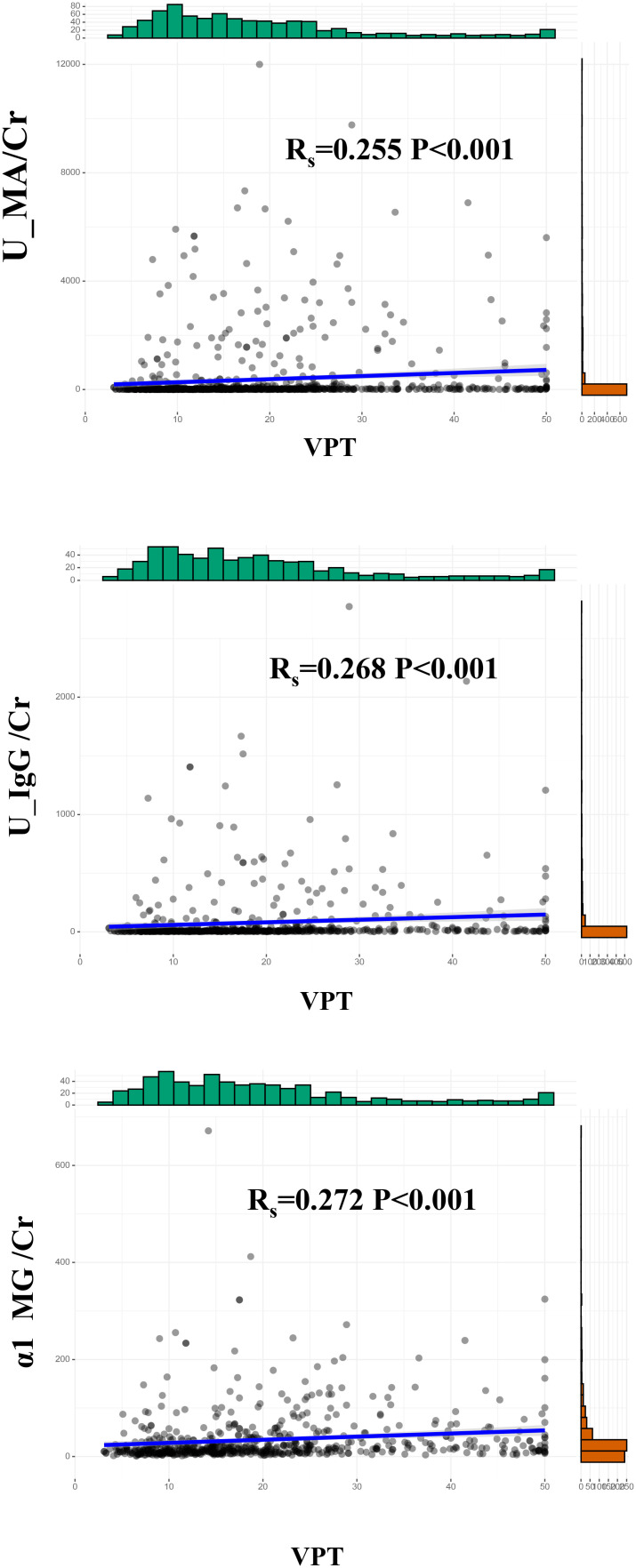
Correlation between VPT and MA/Cr, U-IgG/Cr, andα1-MG/Cr.

**Figure 4:**
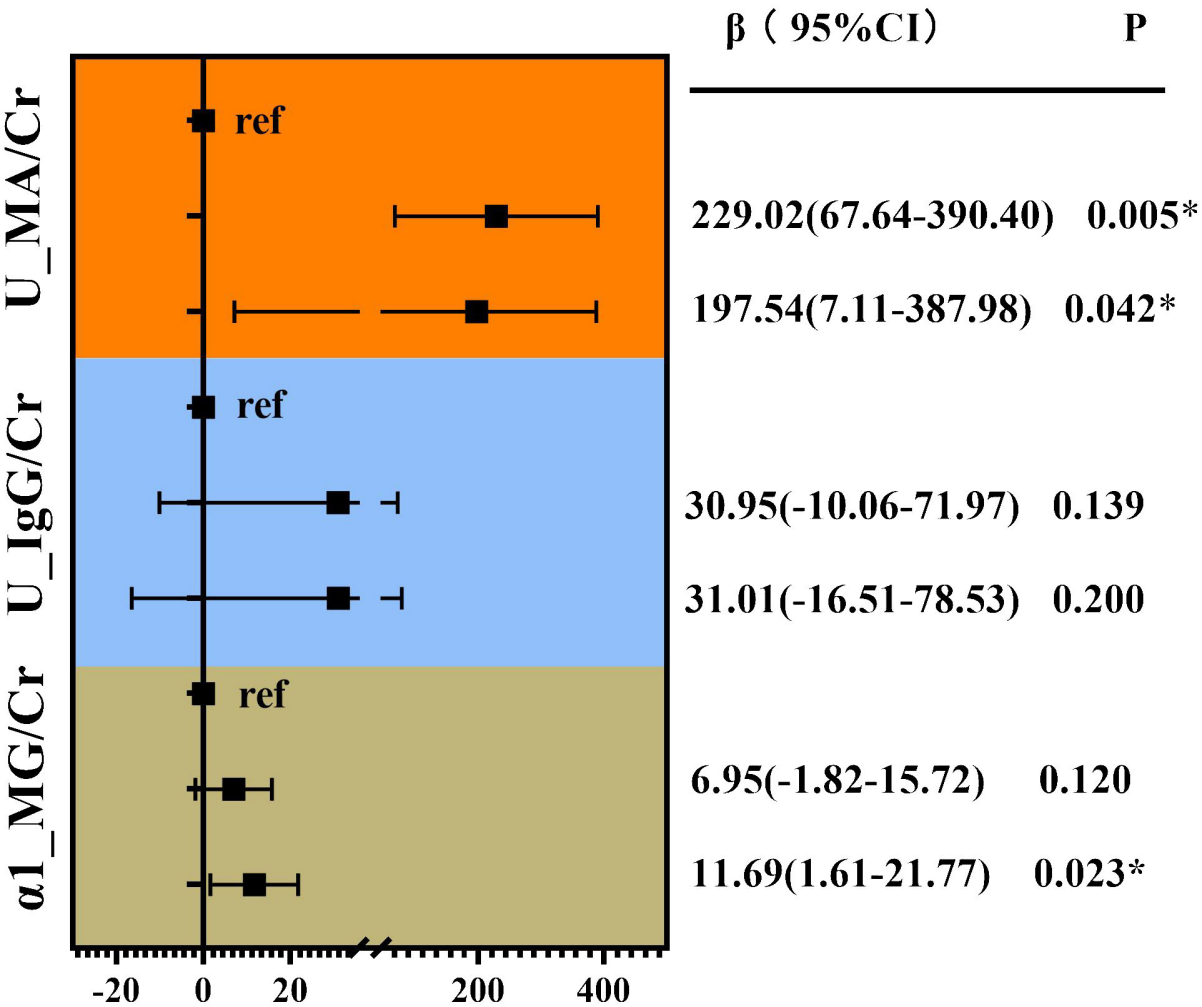
Linear correlation analysis of VPT with glomerular, tubular, and renal artery damage indicators. Adjusted for age, duration of diabetes, family history of diabetes, SBP, DBP, HT, TG, BUN, UA, Cr, history of alcohol consumption, and medication use history. *P<0.05.

Correlation analysis without adjustment showed VPT showed a positive correlation between MA/Cr (Rs=0.255, p<0.001), U-IgG/Cr (Rs=0.268, P<0.001), and α1-MG/Cr levels (Rs=0.272, p<0.001), as shown in **Figure 3**. For the VPT severely elevated group, linear correlation analysis revealed a strong positive correlation between VPT levels and MA/Cr (β=197.54, p=0.042), and α1-MG/Cr levels (β=11.69, p=0.023), after correcting for confounding factors. However, no association was found between VPT and U-IgG/Cr, as shown in **Figure 4**.

## 5 Discussion

The present study aimed to provide a comprehensive understanding of the relationship between VPT and renal function in Chinese patients with T2DM, including glomerular and tubular function indicators. Our findings revealed a positive correlation between VPT and the incidence of CKD, with a stronger association observed between VPT and UACR. Furthermore, VPT was identified as an independent factor associated with tubular and glomerular damage and functional decline.

Chronic complications associated with T2DM often coexist due to shared risk factors such as older age, smoking, longer duration of diabetes, and a history of hypertension and dyslipidemia. The pathogenesis of these complications involves genetic factors, glucose, lipid dysmetabolism, and oxidative stress (21, 22). Both diabetic nephropathy and neuropathy are influenced by hyperglycemia-induced processes such as the formation of advanced glycation end-products, regulation of fatty acids, lipid β-oxidation, mitochondrial dysfunction, and inflammation mediated by lipids (21). The assessment of VPT is a rapid and cost-effective method for evaluating large fiber nerve function, which can help identify individuals with T2DM who may be at risk of foot ulceration and diabetic neuropathy (6).

There is limited knowledge regarding the clinical significance of VPT and its association with the risk of CKD in patients with T2DM. Additionally, the evaluation of renal function solely based on glomerular filtration overlooks significant irreversible damage that can occur in the kidneys, including tubulointerstitial and renal arterial damage. Furthermore, the insidious onset of renal tubulopathy and arteriolopathy adds to the complexity of assessing renal function (23). Therefore, the present study was designed to investigate the correlation between VPT and various aspects of renal function, including glomerular and tubular, in patients with T2DM.

Our findings revealed that the incidence of CKD and UACR >30 mg/g were significantly higher in groups with abnormal VPT, particularly in the severely elevated VPT group. However, after comprehensive adjustment for confounding factors, no significant difference was observed in the incidence of eGFR <60 ml/min between the groups. These results confirm that higher VPT levels are associated with a higher incidence of CKD, with a stronger association observed with UACR levels. Notably, the correlation between eGFR and VPT was found to be weaker compared to the association with UACR, which could be due to several reasons. First, previous research has shown that UACR is a more sensitive indicator of renal function than eGFR in cases of diabetic peripheral neuropathy (24, 25). Second, in the progression of CKD, the increase in UACR and the decrease in eGFR may not necessarily occur in perfect parallel, as eGFR does not have to be reduced when macroalbuminuria is present (24). Lastly, renal hyperfiltration, which occurs in the early stages of CKD, is considered a marker of renal impairment and has been associated with increased urinary albumin excretion (26, 27).

Diabetic nephropathy, the most common form of CKD and ESRD, is typically characterized by diabetic glomerulosclerosis (28). However, in recent years, tubular injury has received increasing attention as a critical mechanism in the development of albuminuria at the early stages of CKD and its contribution to disease progression (29–31). While MA and U-IgG have traditionally served as markers of glomerular damage (32), α1-MG has emerged as an early and specific marker for tubular injury (33). This study demonstrated that VPT could be an independent factor associated with glomerular and tubular injury. The levels of MA and α1-MG displayed transient increases with elevated VPT, and this association persisted even after adjusting for factors such as age, diabetes control parameters, cardiovascular risk factors, and medication usage. Furthermore, the elevated VPT levels observed were associated with a higher prevalence of hypertension (HT) and increased SBP and DBP. This correlation between VPT and glomerular and tubular damage may be attributed to oxidative stress induced by blood pressure fluctuations (34). Oxidative stress can impair mitochondrial function, leading to accelerated β-cell function decline, glucose, and lipid dysmetabolism, ultimately resulting in renal glomerular and tubulointerstitial damage (35).

The present study has some limitations. First, as a single-center study, there may be potential admission rate bias, which could limit the generalizability of the findings. Secondly, due to the lack of a comparison with healthy subjects, the group of ‘non-diabetes comparators’ was used for comparison to address this issue. Since VPT, MA, U-IgG, and α1-MG are not routine tests for non-diabetic patients, this data was unavailable. Third, as a cross-sectional study, it is important to note that causal relationships cannot be inferred from the observed associations between VPT and renal function. To establish causality and better understand the longitudinal impact, further prospective studies are needed. The strengths of the present study are the following: the assessment of VPT was performed, and a comprehensive understanding of renal function, including indicators of glomerular and tubular were screened, providing objective evidence. Furthermore, the sample size was relatively large, further enhancing the credibility of the results.

## 6 Conclusions

Overall, our findings provided further confirmation that higher VPT is an independent factor associated with CKD incidence in patients with T2DM. The correlation between VPT and CKD was found to be particularly strong when considering the level of UACR. Our findings revealed a robust and comprehensive association between higher VPT, glomerular functional decline, and tubular damage. This study expands our understanding of the role of VPT in T2DM and its impact on renal health.

## Data availability statement

The original contributions presented in the study are included in the article/supplementary material. Further inquiries can be directed to the corresponding author.

## Ethics statement

This study was approved by the ethics committee of the First Affiliated Hospital of Fujian Medical University, written informed consent was obtained from the patients: MRCTA, ECFAH of FMU(2017)131.

## Author contributions

YZ: Data curation, Formal analysis, Writing – original draft, Writing – review & editing. BZ: Data curation, Investigation, Writing – original draft. YL: Data curation, Formal analysis, Investigation, Writing – original draft. XS: Conceptualization, Supervision, Writing – review & editing. LH: Conceptualization, Supervision, Writing – review & editing. FZ: Data curation, Investigation, Writing – review & editing. SY: Conceptualization, Data curation, Funding acquisition, Resources, Supervision, Validation, Writing – review & editing.
